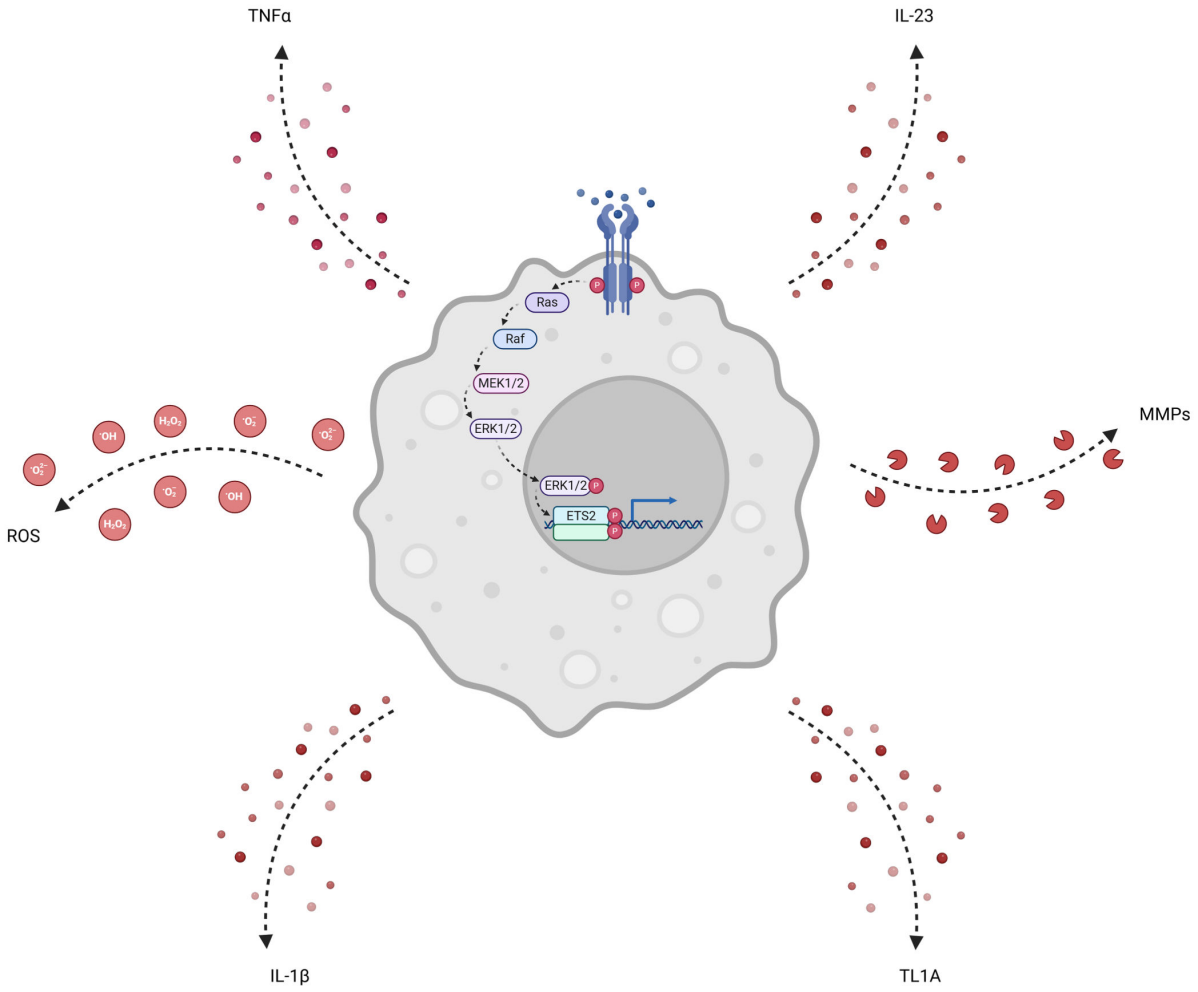# The role of ETS2 in macrophage inflammation

**DOI:** 10.1089/dna.2025.0064

**Published:** 2025-04-14

**Authors:** CT Stankey, JC Lee

**Affiliations:** 1 Genetic Mechanisms of Disease Lab, The Francis Crick Institute, London, UK; 2 Department of Immunology and Inflammation, Imperial College London, UK; 3 Washington University School of Medicine, Saint Louis, MO, USA; 4 Department of Gastroenterology, Royal Free Hospital, London, UK; 5 Institute for Liver and Digestive Health, Division of Medicine, University College London, UK

## Abstract

Autoimmune and inflammatory diseases are rising globally (Conrad et al., 2023; Wu et al., 2023), yet widely effective therapies remain elusive. Most treatments have limited efficacy, significant potential side effects, or eventually lose response, underscoring the urgent need for new therapeutic approaches. We recently discovered that ETS2, a transcription factor, functions as a master regulator of macrophage-driven inflammation – and is causally linked to the pathogenesis of multiple inflammatory diseases via human genetics. The pleotropic inflammatory effects of ETS2 included upregulation of many cytokines that are individually targeted by current disease therapies, including TNFα, IL-23, IL1β and TL1A signalling. With the move towards combination treatment – to maximise efficacy – targeting ETS2 presents a unique opportunity to potentially induce a broad therapeutic effect. However, there will be multiple challenges to overcome since direct ETS2 inhibition is unlikely to be feasible. Here, we discuss these challenges and other unanswered questions about the central role that ETS2 plays in macrophage inflammation.

## Introduction

Autoimmune and inflammatory diseases are characterised by chronic inflammatory responses against host tissues and typically require long-term treatment. However, most drugs only work in a small fraction of patients, or only partially, or eventually stop working, or cause intolerable side effects (Ben-Horin et al., 2022; Gisbert and Panés, 2009; Kennedy et al., 2019). Developing better treatments remains a major unmet need.

Despite this undisputed need, efforts to develop new drugs frequently fail, with consistently high attrition rates observed during clinical trials – most commonly due to a lack of efficacy (Hay et al., 2014; Mullard, 2016). Such failures expose our incomplete understanding of disease mechanisms, with drug candidates that directly target proven disease biology being much more likely to succeed (Morgan et al., 2018). Genetics can provide powerful support, causally linking target genes and pathways to disease pathogenesis. Indeed, drug candidates supported by genetics are over twice as likely to receive approval than those without (King et al., 2019; Minikel et al., 2024; Nelson et al., 2015).

The success of genome-wide association studies (GWAS) therefore provides a unique opportunity to discover better drug targets. GWAS have identified hundreds of susceptibility loci for autoimmune and inflammatory diseases, which have illuminated the genetic architecture of human disease. In a few cases, GWAS findings have directly supported drug development or informed drug repurposing (Sandborn et al., 2008). However, the vast majority of GWAS associations remain poorly understood, principally because most do not alter the coding sequence of genes, but lie in regulatory elements within non-coding genomic regions where their effects are not immediately interpretable.

### A pleiotropic gene desert predisposes to inflammatory disease

By investigating an uncharacterised GWAS association on chromosome 21, which was first linked to disease over 10 years ago, we recently discovered a master regulator of inflammatory macrophages and a central disease pathway that is potentially druggable. This association, which lies within a gene desert and predisposes to five different inflammatory diseases (International Genetics of Ankylosing Spondylitis Consortium (IGAS) et al., 2013; Ji et al., 2017; de Lange et al., 2017; Ortiz-Fernández et al., 2021; Stankey et al., 2024), was shown to contain a monocyte/macrophage-specific super-enhancer, which became stronger in the presence of the risk allele, due to increased binding of a pioneer transcription factor and enhanced chromatin accessibility.

We further showed that the target of this super-enhancer, a distant gene named *ETS2*, was both necessary and sufficient for macrophage inflammatory responses. Disrupting *ETS2* in primary human macrophages profoundly impaired key effector functions and downregulated multiple pathways relating to macrophage activation.

Conversely, overexpressing *ETS2* induced a striking, dose-dependent upregulation of the same pro-inflammatory pathways and induced a pathogenic state that closely resembled the macrophage phenotype seen in diseased tissue from conditions such as inflammatory bowel disease (IBD).

We showed that ETS2 orchestrated macrophage effector responses via distinct mechanisms, including the induction of a metabolic state that was permissive for inflammation and the direct transcriptional control of hundreds of genes within distinct inflammatory processes. Finally, we showed how this might provide a novel therapeutic target; confirming that ETS2-mediated inflammation was evident in diseased tissues and identifying a means to interfere with this pathway using an upstream inhibitor.

### Potential advantages of targeting *ETS2* therapeutically

A major challenge in treating inflammatory diseases is the so-called “therapeutic ceiling”, where most drugs are only effective in a limited proportion of patients (Alsoud et al., 2021). In recent years, the mainstay of advanced therapies have been monoclonal antibodies, which are typically designed to block a single pro-inflammatory molecule (Noor et al., 2024). Recently, it has been shown that combining such drugs can improve response rates (Feagan et al., 2023; Glatt et al., 2018) – most likely because pathological inflammation invariably involves multiple proinflammatory signals (Solitano et al., 2023). Our discovery that ETS2 controls the expression of many proinflammatory cytokines – including several targeted by current therapies – highlights the potential value of targeting ETS2 (Figure 1).

However, like many transcription factors, ETS2 is not a classically druggable target, necessitating approaches other than direct inhibition (Henley and Koehler, 2021; Newman et al., 2015).

### Difficulties for targeted protein degradation approaches

There is growing interest in targeting transcription factors in medicine. One way in which this can be achieved is targeted protein degradation (TPD), where the endogenous ubiquitin-proteasome system is exploited to degrade transcription factors, for example using proteolysis-targeting chimeras (PROTACs) or molecular glues (Békés et al., 2022; Schreiber, 2021). Because these compounds promote interactions with ubiquitin ligases, rather than bind at active or allosteric sites, they can target otherwise undruggable proteins (Tsai et al., 2024). Both approaches are in clinical development, with PROTACs in Phase III trials and molecular glues approved for several indications (Chirnomas et al., 2023).

With increasing data on TPD efficacy, it has become clear that the half-life of the target protein is critical. Indeed, a recent study showed that degraders are minimally effective for proteins with half-lives shorter than 2 hours (Vetma et al., 2024). This poses a major challenge for ETS2 targeting, since the half-life of ETS2 is only 20 minutes (Bhat et al., 1990). A potential alternative could be to suppress *ETS2* at a transcript level through small interfering (si)RNA-or antisense oligonucleotides (ASOs). However, these technologies face challenges related to stability, delivery, immunogenicity, and pharmacokinetics, especially when the target cell is frequently replenished from the bone marrow (Dhuri et al., 2020; Tang and Khvorova, 2024).

### Small molecule drugs modulate ETS2-driven inflammation

Using small molecule drugs to modulate upstream regulators of ETS2 could overcome these challenges. An analogous approach using JAK1/2 inhibitors is known to be effective, preventing activation of STAT transcription factors and suppressing inflammation (van der Heijde et al., 2013; Taylor et al., 2017). We showed that drugs which target the MAPK pathway – which activates ETS-family transcription factors – can downregulate the ETS2 transcriptional programme *in silico* (Stathias et al., 2020) and confirmed that one such class, MEK1/2 inhibitors, which are already used to treat non-inflammatory conditions (de Blank et al., 2022), could reduce pathological inflammation and the secretion of key pro-inflammatory cytokines in IBD biopsies. Strikingly, MEK inhibition decreased inflammatory markers to a similar extent as high-dose infliximab –one of the most effective current therapeutic interventions for IBD (Hahn et al., 2022).

These data suggest that reducing ETS2 signalling via MAPK inhibition could be a tractable therapeutic approach. However, MEK1/2 inhibition may not be the only way in which this could be achieved. We selected MEK1/2 inhibitors for proof-of-concept studies as these were consistently predicted to downregulate *ETS2* signalling *in silico* and because highly selective MEK inhibitors were already approved for clinical use. However, drugs targeting other components of the MAPK pathway were also predicted to reduce ETS2-mediated inflammation. These included RAF and ERK inhibitors – kinases upstream and downstream of MEK respectively. RAF inhibitors are approved in oncology, although their efficacy can be limited by paradoxical MAPK activation, via Ras upregulation leading to increased signalling through the pathway (Hatzivassiliou et al., 2010). ERK inhibitors are currently in clinical trials, but show potent on-target activity and theoretically offer the most direct means to prevent ETS2 activation (Moschos et al., 2018; Sullivan et al., 2018). Studies of these drugs in primary human macrophages will be needed to understand their relative ability to modulate ETS2-driven inflammation.

Although MAPK pathway inhibitors can suppress pathological macrophage inflammation, the essential role of this pathway in other tissues means that they also risk significant on-target side-effects (Subbiah et al., 2020). Selective delivery of these compounds to inflammatory macrophages would be required to facilitate longer-term use in inflammatory diseases. Antibody-drug conjugates (ADCs) offer one method to achieve this. ADCs, which consist of a drug tethered to a monoclonal antibody by a linker, have been approved for treatment of certain cancers and are now in clinical development for immune-mediated diseases (Maecker et al., 2023). ADCs require a cell surface protein that is selectively and highly expressed upon the target cell and, ideally, should facilitate rapid internalisation of the conjugate upon binding. Within the cell, the ADC is cleaved, releasing the payload (drug) (Hasan et al., 2022; Tsuchikama et al., 2024). Preclinical work suggests that monocytes and macrophages are highly amenable to ADC targeting, with selective expression of receptors that facilitate rapid endocytosis (Granfeldt et al., 2013; Howe et al., 2023). Whether these could be used to deliver a sufficient amount of a MAPK pathway inhibitor to modulate ETS2-driven inflammation will need to be tested.

### Gene-environment interactions at the chr21q22 locus

In addition to uncovering new therapeutic targets, a fundamental goal of GWAS was to generate biological insights into disease. Methods to identify links between genetic variation and chromatin state, transcript levels, or other cellular phenotypes provide an important starting point, but understanding the underlying biology often requires more in-depth investigation. For instance, while the disease-associated chr21q2 locus was known to increase *ETS2* expression in macrophages, the importance and mechanistic basis of ETS2-driven inflammation were previously undiscovered.

Studying genetics can also provide insights into broader aspects of biology. For example, we were intrigued as to why the risk allele at chr21q22 remained so common globally despite conferring susceptibility to multiple inflammatory diseases. Recent studies have not suggested a rapid spread of the risk allele (a “strong selective sweep”) over the past few thousand years (Irving-Pease et al., 2024; Le et al., 2022), which led us to investigate when the risk allele first arose in humans.

Remarkably, we found that the causal variant was over 500,000 years old, and was polymorphic even in Neanderthals and Denisovans – thus having been present in humans ever since the origin of species. We reasoned that this pathway must confer some beneficial effect under certain circumstances; to explain why it had not been lost via natural selection. This led us to investigate other situations in which macrophage-driven inflammation occurs, and discover that the genetic basis of inflammatory disease risk was exactly the same as the genetic basis for increased *ETS2* expression following early exposure to bacteria. Further investigation revealed that the transcriptional state of macrophages during serious bacterial infections is significantly enriched for genes induced by ETS2. The time following first exposure to bacteria is a critical period in which effective pathogen killing and clearance – via processes such as reactive oxygen species production, phagocytosis, and cytokine production – can prevent disseminated infection. Since ETS2 regulates all of these processes, this would provide a strong selective pressure to maintain the inflammatory disease risk allele in the population, and usefully indicates that any goal of ETS2-directed therapy should be to lessen signalling, not abrogate it entirely.

### ETS2 in other disease contexts

Aside from essential roles in inflammatory disease and bacterial infection, it is possible that ETS2 is involved in other human diseases. For example, although ETS2-driven inflammation was not enriched in classical tumour-associated macrophages, which are typically anti-inflammatory and contribute to the immunosuppressive tumour microenvironment, some tumours are characterised by an influx of inflammatory monocyte-derived macrophages which may contribute to cancer development and spread (Caronni et al., 2023; Hill et al., 2023). These TAMs are induced in response to signals that upregulate *ETS2*, including TNFα and prostaglandin E2, and express high levels of cytokines induced by ETS2, such as IL1β (Xue et al., 2014). Whether *ETS2* directly contributes to this process warrants additional investigation. Similarly, whether the extra copy of ETS2 might contribute to the low-grade inflammation observed in Trisomy 21 (Down syndrome) is also worth exploring, especially since this could be targeted therapeutically.

## Conclusion

Genetics can help elucidate disease mechanisms, provide insights into fundamental biology and identify new drug targets, which are especially valuable when the associations predispose to several diseases. However, even when this is endeavour is successful – as in the case of *ETS2* – there remain many unanswered questions.

The multi-hit model of complex disease development requires hits in several key pathways, most of which remain unknown. Similarly, how genetic risk interacts with poorly-characterised environmental triggers is not understood, despite the global increases in disease incidence being directly attributable to this process. With regard to ETS2, the discovery of a feed-forward loop – whereby ETS2 binds to its own super-enhancer to further amplify its expression – means that as-yet-uncharacterised inhibitory mechanisms must exist to facilitate a return to homeostasis. Answering these questions, and elucidating the mechanisms involved, should provide a better understanding of disease biology, generate new insights into fundamental immunological processes and reveal novel therapeutic targets.

## Figures and Tables

**Figure:**